# Immunoprophylaxis against Mother-to-Child Transmission of HIV-1

**DOI:** 10.1371/journal.pmed.0030259

**Published:** 2006-07-18

**Authors:** Miroslaw K Gorny, Susan Zolla-Pazner

## Abstract

The authors discuss a new study in *PLoS Medicine* that examines whether human anti-HIV monoclonal antibodies might be useful in South Africa for preventing mother-to-child transmission of subtype C HIV viruses.

In resource-limited settings, there is a high risk of mother-to-child-transmission (MTCT) of HIV-1. The rate of MTCT without specific intervention and with an extended time of breast-feeding is about 35%–40% [ 1], a figure that results in the infection of about 750,000 children every year worldwide [ 2]. A single dose of nevirapine can reduce the rate of MTCT by 42%, but it selects for drug-resistant variants in as many as 75% of mothers receiving this treatment [ 3]. As multiple-drug treatment is rarely available in developing countries, and breast-feeding often continues for at least two years after delivery, the concept of preventing MTCT transmission by passive administration of antibodies, by the use of antibodies and drugs, or by the use of combined active–passive immunization is attracting increasing attention [ 4].

## Studies of Passive Immunization

Passive immunization experiments have proven that antibodies can protect against HIV-1 infection in animal models. Polyclonal or monoclonal antibodies (mAbs) against simian immunodeficiency virus (SIV) or HIV-1 have mediated protection of chimpanzees from HIV-1 infection [ 5] and protection of juvenile and neonatal macaques from infection with SIV or with chimeric simian/human immunodeficiency virus (SHIV) [ 6–10]. In several experiments with SHIV89.6P, broadly neutralizing human mAbs b12, 2G12, 2F5, and 4E10 were tested [ 6, 7, 9, 10]. These mAbs were generated from subtype-B-infected individuals. Although HIV-1 subtype B is the predominant subtype in North America, Western Europe, and Australia, HIV-1 subtype B viruses account for only about 12% of the global HIV pandemic [ 11]. The mAbs tested in these experiments react with epitopes in the CD4 binding domain of gp120 (mAb b12), with gp120 glycans (mAb 2G12), and with epitopes in the membrane proximal region of gp41 (mAbs 2F5 and 4E10). A combination of these mAbs provided a stronger protective effect than any of the single mAbs, which individually mediated only partial protection at best against chimeric simian/human immunodeficiency virus [ 6].

There is a critical need to determine if passive immunization will decrease mother-to-child transmission of HIV.

The evidence for the function of anti-HIV-1 antibodies in preventing MTCT in humans is less conclusive than in animal models. Early studies showed a positive correlation between the presence of neutralizing antibodies in mothers and lower incidence of MTCT [ 12, 13], although more recently these findings have not been replicated [ 14]. Moreover, there are few data on the effectiveness of passive immunization in the prevention of MTCT in humans. Only one study reported the use in HIV-infected pregnant women of HIVIG, an immune globulin preparation from HIV-infected individuals containing high levels of anti-HIV-1 antibodies. This clinical trial was inconclusive because of the overall low transmission rate in the study groups, but it showed an intriguing trend toward lower transmission with HIVIG than with control immunoglobulin [ 15].

## A New mAb Study

In a new paper published in *PLoS Medicine*, Gray et al. address the question of whether human anti-HIV mAbs 2F5, 2G12, b12, and 4E10 might be useful in South Africa as reagents to prevent MTCT of subtype C viruses [ 16]. To examine this question, they tested these mAbs, which had previously been used in monkey passive immunization studies (see above), for their ability to neutralize in vitro seven subtype C primary isolates from pediatric patients. The study shows that two of the mAbs, 2G12 and 2F5, had no neutralizing activity against the subtype C isolates tested. The authors conclude that these two mAbs should not proceed into passive immunization clinical trials in southern Africa and other regions where HIV-1 subtype C viruses predominate. Of the other two mAbs tested, b12 and 4E10, the former was shown to be potent, with a 50% neutralizing dose in the range of 0.2 to 11.9 μg mAb/ml, but capable of neutralizing only four of the seven isolates; in contrast, 4E10 lacked potency, requiring up to 46 μg mAb/ ml to achieve 50% neutralization, but it eventually neutralized all of the isolates tested.

Although mAbs b12 and 4E10 did display neutralizing activity against subtype C viruses, these mAbs have recently been shown to have the characteristics of polyspecific and auto-reactive antibodies. Thus, mAb b12 recognizes the CD4 binding site of gp120 as well as ribonucleoproteins, double-stranded DNA, centromere B antigens, histones, and cytoplasmic and nucleolar antigens of HEp-2 cells. Similarly, mAb 4E10 recognizes an epitope in the membrane proximal region of gp41 and also binds to host antigens including cardiolipin, phospholipids, lupus erythematosus autoantigen SS-A/Ro, and cytoplasmic and nuclear antigens of HEp-2 cells, and has lupus anticoagulant reactivity [ 17]. Crystallographic analysis of the complex of the Fab fragment of 4E10 with gp41 peptide showed that the CDR H3 has no contact with the gp41 peptide [ 18], suggesting that the CDR H3, which is the major loop of the antibody binding site involved in the interaction with antigen, might interact with membrane components such as phospholipids.

To date, there are no published data that suggest that these particular mAbs with autoreactive activity are pathogenic. Indeed, mAbs 4E10, 2G12, and 2F5 were tested as reagents for passive immunotherapy in HIV-infected individuals and no toxicity was observed [ 19]. mAb 4E10, however, did not reach the same plasma level as mAb 2G12 in this clinical trial and did not induce escape mutants as did 2G12, suggesting that mAb 4E10 could have been absorbed out by host antigens [ 19]. Concern is compounded by the observation that anti-cardiolipin antibodies are associated with thrombosis and procoagulopathies [ 20], indicating that the risk assessment for antibodies with autoreactive characteristics must be stringent, especially when use in infants is contemplated.

## Clinical Implications

In summary, the accumulated data suggest that although antibodies have a role in preventing or decreasing the rate of MTCT of HIV-1, the human anti-HIV mAbs currently available will not suffice as reagents for passive immunization in most parts of the developing world, where subtype B viruses (the subtype most effectively targeted by most of the currently available human mAbs) are rare. Currently, there is a critical need to determine if passive immunization with HIVIG will definitively decrease MTCT, and if so, there needs to be a concerted effort to develop the specific mAbs to be used alone, in combination, or together with antiretroviral drugs to prevent the infection of infants.

## Abbreviations

mAb: monoclonal antibody
MTCT: mother-to-child-transmission

## References

[pmed-0030259-b1] Newell ML (2006). Current issues in the prevention of mother-to-child transmission of HIV-1 infection. Trans R Soc Trop Med Hyg.

[pmed-0030259-b2] Joint United Nations Programme on HIV/ AIDS (2002). Report on the global HIV/ AIDS epidemic: 2002.

[pmed-0030259-b3] Smith DM (2006). The controversies of nevirapine for preventing mother-to-child HIV transmission. AIDS.

[pmed-0030259-b4] Safrit JT, Ruprecht R, Ferrantelli F, Xu W, Kitabwalla M (2004). Immunoprophylaxis to prevent mother-to-child transmission of HIV-1. J Acquir Immune Defic Syndr.

[pmed-0030259-b5] Emini EA, Schleif WA, Nunberg JH, Conley AJ, Eda Y (1992). Prevention of HIV-1 infection in chimpanzees by gp120 V3 domain-specific monoclonal antibody. Nature.

[pmed-0030259-b6] Mascola JR, Lewis MG, Stiegler G, Harris D, VanCott TC (1999). Protection of macaques against pathogenic simian/human immunodeficiency virus 89.6PD by passive transfer of neutralizing antibodies. J Virol.

[pmed-0030259-b7] Baba TW, Liska V, Hofmann-Lehmann R, Vlasak J, Xu W (2000). Human neutralizing monoclonal antibodies of the IgG1 subtype protect against mucosal simian-human immunodeficiency virus infection. Nat Med.

[pmed-0030259-b8] Van Rompay KK, Berardi CJ, Dillard-Telm S, Tarara RP, Canfield DR (1998). Passive immunization of newborn rhesus macaques prevents oral simian immunodeficiency virus infection. J Infect Dis.

[pmed-0030259-b9] Hofmann-Lehmann R, Vlasak J, Rasmussen RA, Smith BA, Baba TW (2001). Postnatal passive immunization of neonatal macaques with a triple combination of human monoclonal antibodies against oral simian-human immunodeficiency virus challenge. J Virol.

[pmed-0030259-b10] Ferrantelli F, Rasmussen RA, Buckley KA, Li PL, Wang T (2004). Complete protection of neonatal rhesus macaques against oral exposure to pathogenic simian-human immunodeficiency virus by human anti-HIV monoclonal antibodies. J Infect Dis.

[pmed-0030259-b11] Osmanov S, Pattou C, Walker N, Schwardlander B, Esparza J (2002). Estimated global distribution and regional spread of HIV-1 genetic subtypes in the year 2000. J Acquir Immune Defic Syndr.

[pmed-0030259-b12] Rossi P, Moschese V, Broliden PA, Fundaro C, Quinti I (1989). Presence of maternal antibodies to human immunodeficiency virus 1 envelope glycoprotein gp120 epitopes correlates with the uninfected status of children born to seropositive mothers. Proc Natl Acad Sci U S A.

[pmed-0030259-b13] Scarlatti G, Albert J, Rossi P, Hodara V, Biraghi P (1993). Mother-to-child transmission of human immunodeficiency virus type. 1: Correlation with neutralizing antibodies against primary isolates. J Infect Dis.

[pmed-0030259-b14] Guevara H, Casseb J, Zijenah LS, Mbizvo M, Oceguera LF (2002). Maternal HIV-1 antibody and vertical transmission in subtype C virus infection. J Acquir Immune Defic Syndr.

[pmed-0030259-b15] Stiehm ER, Lambert JS, Mofenson LM, Bethel J, Whitehouse J (1999). Efficacy of zidovudine and human immunodeficiency virus (HIV) hyperimmune immunoglobulin for reducing perinatal HIV transmission from HIV-infected women with advanced disease: Results of Pediatric AIDS Clinical Trials Group protocol 185. J Infect Dis.

[pmed-0030259-b16] Gray ES, Meyers T, Gray G, Montefiori D, Morris L (2006). Insensitivity of paediatric HIV-1 subtype C viruses to broadly neutralizing monoclonal antibodies raised against subtype B. PLoS Med.

[pmed-0030259-b17] Haynes BF, Fleming J, St Clair WE, Katinger H, Stiegler G (2005). Cardiolipin polyspecific autoreactivity in two broadly neutralizing HIV-1 antibodies. Science.

[pmed-0030259-b18] Cardoso RM, Zwick MB, Stanfield RL, Kunert R, Binley JM (2005). Broadly neutralizing anti-HIV antibody 4E10 recognizes a helical conformation of a highly conserved fusion-associated motif in gp41. Immunity.

[pmed-0030259-b19] Trkola A, Kuster H, Rusert P, Joos B, Fischer M (2005). Delay of HIV-1 rebound after cessation of antiretroviral therapy through passive transfer of human neutralizing antibodies. Nat Med.

[pmed-0030259-b20] Erkan D, Lockshin MD (2006). Antiphospholipid syndrome. Curr Opin Rheumatol.

